# Fluralaner (Bravecto^®^) treatment kills *Aedes aegypti* after feeding on *Dirofilaria immitis*-infected dogs

**DOI:** 10.1186/s13071-023-05819-9

**Published:** 2023-06-20

**Authors:** Kathryn Duncan, Anne W. Barrett, Susan E. Little, Kellee D. Sundstrom, Frank Guerino

**Affiliations:** 1 Merck Animal Health, Rahway, NJ USA; 2 College of Veterinary Medicine, Oklahoma State University, Stillwater, OK USA

**Keywords:** *Aedes*, *Dirofilaria*, Fluralaner, Heartworm, Isoxazoline, Mosquito

## Abstract

**Background:**

Transmission of canine heartworm (*Dirofilaria immitis*) from infected to naïve dogs is dependent on successful mosquito feeding and survival.

**Methods:**

To determine whether treating heartworm-infected dogs with fluralaner (Bravecto^®^) limits the survival of infected mosquitoes, and potentially the transmission of *D. immitis*, we allowed female mosquitoes to feed on microfilaremic dogs and evaluated mosquito survival and infection with *D. immitis*. Eight dogs were experimentally infected with *D. immitis*. On day 0 (~ 11 months post-infection), four microfilaremic dogs were treated with fluralaner according to label directions while the other four were non-treated controls. Mosquitoes (*Aedes aegypti* Liverpool) were allowed to feed on each dog on days −7, 2, 30, 56, and 84. Fed mosquitoes were collected, and the number of live mosquitoes determined at 6 h, 24 h, 48 h, and 72 h post-feeding. Surviving mosquitoes held for 2 weeks were dissected to confirm third-stage *D. immitis* larvae; PCR (12S rRNA gene) was performed post-dissection to identify *D. immitis* in mosquitoes.

**Results:**

Prior to treatment, 98.4%, 85.1%, 60.7%, and 40.3% of mosquitoes fed on microfilaremic dogs were alive at 6 h, 24 h, 48 h, and 72 h post-feeding, respectively. Similarly, mosquitoes fed on microfilaremic, non-treated dogs were alive 6 h post-feeding (98.5–100%) throughout the study. In contrast, mosquitoes fed on fluralaner-treated dogs 2 days after treatment were dead or severely moribund by 6 h post-feeding. At 30 and 56 days post-treatment, > 99% of mosquitoes fed on treated dogs were dead by 24 h. At 84 days post-treatment, 98.4% of mosquitoes fed on treated dogs were dead by 24 h. Before treatment, third-stage larvae of *D. immitis* were recovered from 15.5% of *Ae. aegypti* 2 weeks after feeding, and 72.4% were positive for *D. immitis* by PCR. Similarly, 17.7% of mosquitoes fed on non-treated dogs had *D. immitis* third-stage larvae 2 weeks after feeding, and 88.2% were positive by PCR. Five mosquitoes fed on fluralaner-treated dogs survived 2 weeks post-feeding, and 4/5 were from day 84. None had third-stage larvae at dissection, and all were PCR-negative.

**Conclusion:**

The data indicate that fluralaner treatment of dogs kills mosquitoes and thus would be expected to reduce transmission of heartworm in the surrounding community.

**Graphical Abstract:**

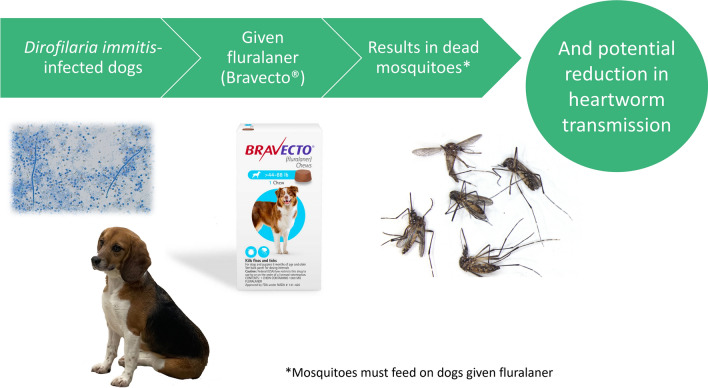

**Supplementary Information:**

The online version contains supplementary material available at 10.1186/s13071-023-05819-9.

## Background

Fluralaner is an isoxazoline insecticide and acaricide registered for use in the United States (USA) under the trade name Bravecto^®^ (Merck Animal Health, Rahway, NJ, USA) as an ectoparasiticide for the treatment and control of fleas and ticks on dogs for up to 12 weeks [1–3]. After oral or transdermal administration, fluralaner activity remains high, with experimental efficacy against ticks and fleas recorded for as long as 3.8 and 4 months, respectively, and for preventing transmission of some vector-borne diseases [4–7]. Additionally, fluralaner is a highly effective, persistent insecticide and acaricide with documented efficacy against mites and lice as well as in vitro efficacy against mosquitoes [8–12]. Besides its ability to significantly reduce mosquito survival for up to 12 weeks in a laboratory setting, fluralaner has been shown to suppress egg-laying of any surviving mosquitoes [13]. However, the mosquitocidal effect of direct feeding on fluralaner-treated dogs has not been reported.

Mosquitoes are required as intermediate hosts and vectors for *Dirofilaria immitis*, the causative agent of heartworm disease. Surveys have documented that more than 20 species of mosquitoes in North America have been found to have infective third-stage larvae of *D. immitis* and therefore may be competent vectors of this pathogen to dogs [14, 15]. Canine heartworm disease (CHD) is a significant threat to dogs across the globe, and unfortunately, the prevalence of *D. immitis* infection appears to have been increasing over the past decade [16, 17]. The relocation or translocation of infected dogs, emergence of macrocyclic lactone-resistant isolates, and the continued biogeographical changes across its range all appear to play a role in the expansion of heartworm [17–20]. For instance, climate modeling in Europe demonstrates that summers are likely warm enough—even in high-altitude areas—for *Dirofilaria* spp. transmission which could lead to expansion to historically heartworm-free regions [18]. These changes may be responsible, in part, for the introduction and establishment of *D. immitis*-competent invasive species, such as *Aedes albopictus*, which is thought to be important in the changing geographic patterns of heartworm [15]. In North America, mosquito populations have also expanded in recent decades, and as suburban or urban expansion continues, peridomestic mosquitoes capable of transmitting heartworm, such as *Aedes aegypti*, may be given the opportunity to flourish [15, 21]. Therefore, new strategies are needed to limit *D. immitis* transmission in endemic areas.

Isoxazolines have been proposed as a means to limit transmission of mosquito-borne disease agents in other regions [11]. Therefore, when heartworm-positive dogs are given fluralaner, the mosquitocidal effects may reduce the population of *D. immitis*-infected mosquitoes and limit transmission of heartworm from infected individuals to non-infected individuals. Here, we present the results of a good clinical practice (GCP) laboratory experiment which evaluated mosquitoes after feeding on heartworm-infected dogs administered a single oral dose of fluralaner at the labeled-approved dose. We allowed mosquitoes to feed on microfilaremic dogs and evaluated mosquito survival, mosquito infection with *D. immitis*, and development of *D. immitis* third-stage larvae.

## Methods

### Animals

Eight approximately 2-year-old spayed female laboratory-reared beagles were purchased from a commercial supplier, and upon receipt were deemed healthy by physical examination and negative for both heartworm antigen and microfilaria when tested with a commercial antigen test (DiroCHEK^®^, Zoetis Animal Health, New Jersey, USA) according to the manufacturer’s instructions and modified Knott test, respectively [22]. All dogs were housed indoors at Oklahoma State University’s AAALAC (Association for Assessment and Accreditation Laboratory Animal Care)-accredited laboratory animal facilities and cared for by Oklahoma State University’s laboratory animal care staff. Throughout the study, standard care protocols were followed which were approved by the Oklahoma State University Institutional Animal Care and Use Committee (IACUC). General health observations began within 1 week of animals arriving at the facility and continued daily on weekdays until the end of the study.

### *Dirofilaria immitis* inoculation and heartworm testing

On the day of inoculation, third-stage *D. immitis* larvae (Berkeley isolate, TRS Labs, Inc., Athens, GA, USA) were harvested from *Ae. aegypti* mosquitoes (Liverpool strain) which had been infected by artificially feeding on microfilaremic blood as previously described [23]. Immediately following this, larvae (*n* = 50) were subcutaneously injected into the left inguinal region of each dog. No adverse events were noted following inoculation. Blood collection began 84 days post-infection (dpi) for antigen and microfilariae testing and was continued weekly until infection was confirmed. Similar to the initial heartworm screening, a commercial antigen test (DiroCHEK^®^, Zoetis Animal Health, New Jersey, USA) was used according to the manufacturer’s instructions, and a modified Knott test was utilized for microfilaria testing [22]. Additionally, all antigen tests were performed on serum with or without heat reversal treatment [24] and further evaluated using a spectrophotometer (BioTek Cytation 5, Agilent, Santa Clara, CA, USA) to determine optical density (OD) readings. To standardize all OD values across multiple plates, the negative control OD reading value was subtracted from each sample’s OD reading according to plate and then recorded. Testing also occurred approximately 1 day prior to each mosquito challenge to confirm positive heartworm infection using the methods described above.

### Treatment groups

The eight dogs were evenly allocated into two groups (treatment or control). Seven of the dogs were randomly assigned to groups using a randomized complete block design with microfilaria counts as the blocking factor; a single dog which remained amicrofilaremic throughout the entirety of the study was placed deliberately into the control group to support the study objective and maximize the likelihood of microfilaria ingestion by mosquitoes fed on treated dogs. More information on the amicrofilaremic dog can be found in the subsequent sections. The two groups were housed in separate rooms, and dogs within a treatment group were co-housed in pairs for enrichment. To avoid cross-contamination, all socialization of dogs was conducted within treatment groups, personal protective equipment (PPE) was changed upon exiting each room, and all equipment was assigned to a particular group and remained there until the end of the study. Dogs were weighed 1 day prior to treatment administration. On day 0, approximately 11 months (330 days) since inoculation with *D. immitis* third-stage larvae, fluralaner (Bravecto^®^ 13.64% w/w fluralaner flavored chewable tablets for dogs) was administered to dogs in the treatment group in a single oral dose according to label recommendations based on each dog’s individual weight and within 20 min of receiving food. None of the treated dogs regurgitated or displayed any adverse reactions following treatment. At the time of treatment administration, sham doses (single food kibble) were given to control dogs within 20 min of receiving food. Personnel involved with general health observations, mosquito challenges and evaluations, sample collections and processing, and analyses were masked to treatment assignments for each group.

### Mosquito challenges

Recently emerged female mosquitoes (*Ae. aegypti* Liverpool) were obtained from Benzon Research (https://www.benzonresearch.com/home) approximately 1–7 days before each mosquito challenge. Upon receipt, they were stored in individual cups (approximately 50 mosquitoes per cup) in an insectary under standard conditions (27 °C ± 1 °C, relative humidity 80% ± 5%, 12-h light/dark photoperiod) [25]. Mosquitoes were provided a 10% sucrose diet at all times except when fasted on water alone approximately 12 h (h) before each challenge. Mosquito challenges occurred on days −7, 2, 30, 56, and 84. Immediately prior to each mosquito challenge, dogs were weighed and then sedated with intramuscular dexmedetomidine hydrochloride (DEXDOMITOR^®^, Zoetis Inc., Kalamazoo, MI, USA) according to label directions. Because some dogs experienced moderate insect hypersensitivity (facial swelling) after the first mosquito challenge, all dogs were pretreated with injectable diphenhydramine hydrochloride (Armas Pharmaceuticals, Inc., Freehold, NJ, USA) at 1 mg/kg intramuscularly at each subsequent challenge as recommended by a case report of canine mosquito bite hypersensitivity [26]. Dogs were then placed individually in a mesh-lined enclosure where approximately 100 *Ae. aegypti* were released and given approximately 60 min to feed. Live and moribund mosquitoes were collected using gentle aspiration; live mosquitoes were defined as those exhibiting normal post-feeding behavior such as resting on the sides of the enclosure or flying without dysfunction, while moribund mosquitoes were characterized by their lethargic behavior at the bottom of the enclosure or inability to fly properly [25]. The numbers of live fed, live unfed, and dead mosquitoes from each enclosure were counted, and live fed mosquitoes were retained for further evaluation, but dead/crushed or unfed mosquitoes were not evaluated for the remainder of the study. After all mosquitoes were collected, sedation was reversed with atipamezole hydrochloride (ANTISEDAN^®^, Zoetis Inc., Kalamazoo, MI, USA) according to label directions.

### Evaluation of mosquito mortality

Fed mosquitoes which were either live or moribund were returned to the insectary where they were held, grouped by the dog on which they fed. Each group was evaluated at 6, 24, 48, and 72 h after feeding, and the number of dead and live mosquitoes determined by visual inspection. Any surviving mosquitoes after 72 h were maintained for an additional 11 days (2 weeks post-feeding).

### Detection of *Dirofilaria immitis* larvae in mosquitoes

The surviving mosquitoes, which were held for 2 weeks following feeding, were dissected as previously described to isolate and count third-stage *D. immitis* larvae, if present [27]. Nucleic acids (DNA) from each mosquito were extracted individually using a commercial kit (DNeasy Blood Kit; Qiagen, Hilden, Germany), and a subset of mosquitoes (*n* = 23) were further evaluated by dividing each specimen into head and thorax/abdomen prior to extraction. To identify the presence of any stage of *D. immitis* in mosquitoes, polymerase chain reaction (PCR) was performed on each nucleic acid sample to amplify a ~ 330-base-pair (bp) fragment of the filarioid mitochondrial 12S ribosomal RNA (rRNA) gene; validated primers (Fila12SF: 5′-CGGGAGTAAAGTTTTGTTTAAACCG-3′ and Fila12SR: 5′-CATTGACGGATGGTTTGTACCAC-3′) were utilized [28]. Electrophoresis on a 2% agarose gel was used to confirm product amplification.

### Statistical analyses

All descriptive statistics (mean, average, range, standard deviation, proportion, and exact binomial 95% confidence interval [CI]) were calculated using Microsoft Excel (Microsoft Office version 2202). For both groups at each mosquito challenge (2, 30, 56, and 84 days after treatment), the arithmetic mean was calculated from live mosquito counts at 6, 24, 48, and 72 h after feeding, and percent survival of live mosquitoes was calculated using these means. Percent reduction (100 − percent survival) was determined for mosquitoes fed on fluralaner-treated dogs. Using the Microsoft Excel Data Analysis ToolPak (Microsoft Office Version 2202), paired *t*-tests were performed on live mosquito mean counts between control and fluralaner-treated dogs. The level of significance was set at α = 0.05. A single control dog which remained microfilaria-negative throughout the study was excluded from all calculations and comparisons, as the lack of microfilariae may have affected mosquito survival; data from this dog have been included in the manuscript to report the occult finding and document the effect of high microfilarial load on the survival of mosquitoes.

## Results

### Heartworm testing results

All dogs were antigen-positive by 147 dpi after heat reversal treatment of serum, while seven of the dogs were antigen-positive by 168 dpi without heat reversal treatment of serum. A single dog took 42 extra days (210 dpi) to become antigen-positive without heat reversal treatment of serum. By 189 dpi, seven of the dogs became microfilaremic. A single dog remained amicrofilaremic throughout the entire study, and to be clear, this is not the dog that took extra time to become antigen-positive without heat reversal mentioned above. Before a mosquito challenge, all dogs were *D. immitis* antigen-positive based on the OD readings of the antigen results before and after heat reversal treatment of serum (Table 1). Before the mosquito challenges, there was a range, on average, of 18,512.5 (± 14,032.8) to 32,410.0 (± 20,933.2) microfilariae/ml of blood (Table 1). More specifically, microfilariae counts of the seven microfilaremic dogs ranged from 15,150 to 55,660 microfilariae/ml per dog.

**Table 1:** Average group results with standard deviations of *Dirofilaria immitis* antigen testing using DiroCHEK^®^ (Zoetis Animal Health, New Jersey, USA)—optical density (OD) readings were obtained at the time of result interpretation for both pre-heat and post-heat treatment of samples—and microfilaria testing (microfilaria [mf] per ml of blood) which occurred before each mosquito challenge

Study day		Average antigen OD^a^		Average mf/ml^c^
		Pre-heat treatment	Post-heat treatment	
−8				
	Control	0.25 ± 0.11	0.93 ± 0.39	18,383.3 ± 4776.8
	Fluralaner	0.18 ± 0.12	0.78 ± 0.07	20,215.0 ± 3.530.4
1				
	Control	NA^b^	NA^b^	NA^b^
	Fluralaner	NA^b^	NA^b^	NA^b^
29				
	Control	0.22 ± 0.12	0.70 ± 0.11	23,306.7 ± 6595.3
	Fluralaner	0.32 ± 0.12	0.65 ± 0.07	18,512.5 ± 14,032.8
55				
	Control	0.15 ± 0.04	0.76 ± 0.05	30,606.7 ± 10,717.2
	Fluralaner	0.18 ± 0.10	0.71 ± 0.14	30,285.0 ± 18,255.8
83				
	Control	0.33 ± 0.10	0.92 ± 0.07	32,410.0 ± 20,933.2
	Fluralaner	0.44 ± 0.15	0.93 ± 0.03	21,750.0 ± 13,970.6

*NA* not applicable

^a^Negative control value subtracted from each sample OD reading before averages were calculated

^b^ Testing was not performed since results were obtained less than 10 days earlier

^c^Only three of the four control dogs became microfilaremic; the amicrofilaremic dog was excluded from these calculations. For more information on the occult dog, please see the Discussion

### Survival of mosquitoes fed on non-treated, microfilaremic dogs

Mosquitoes fed on microfilaremic dogs prior to treatment (day −7 challenge) had mean percent survival of 98.4% (95% CI 97.0–99.2), 85.1% (95% CI 82.1–87.7), 60.7% (95% CI 56.8–64.5), and 40.3% (95% CI 36.5–44.2) at 6 h, 24 h, 48 h, and 72 h post-feeding, respectively, establishing the proportion of mosquitoes that survived through collection, feeding, incubation, and high microfilaria load. For challenges following treatment (days 2, 30, 56, and 84), the mean number of mosquitoes which were alive after feeding on non-treated controls ranged from 86.0–94.7 6 h post-feeding, 63.3–69.3 24 h post-feeding, 13.3–33.7 48 h post-feeding, and 8.7–24.3 72 h post-feeding (Table 2). Mean live counts and percent survival of mosquitoes gradually reduced post-feeding, but regardless of mosquito challenge/days post-treatment, > 98% of mosquitoes that fed on non-treated dogs were alive 6 h post-feeding (Table 3). At 24 h post-feeding, 66.2–78.4% of the mosquitoes were alive; by 48 h post-feeding, 14.3–39.0% were alive; and by 72 h post-feeding, 9.1–28.2% were alive (Table 3). When mosquitoes were assessed for survival 2 weeks after feeding on non-treated microfilaremic dogs, 19–39 mosquitoes were alive from the day −7 challenge, but otherwise, a range of 7–12 mosquitoes survived 2 weeks post-feeding from the other four challenges (Table 4).

**Table 2:** Mean (arithmetic) counts of live mosquitoes 6 h (h), 24 h, 48 h, and 72 h after feeding on non-treated (*n* = 3)^a^ and fluralaner-treated (*n* = 4) heartworm-infected, microfilaremic dogs

Days after treatment	Live, fed mosquitoes	6 h	24 h	48 h	72 h
2					
Control	95.7	94.7	63.3	13.7	8.7
Fluralaner	101.3	0	0	0	0
*P*-value		< 0.0001^b^	< 0.0001^b^	0.0009^b^	0.0225^b^
30					
Control	90.7	89.3	69.3	13.3	11.0
Fluralaner	85.0	3.3	0.5	0.3	0.3
*P*-value		< 0.0001^b^	< 0.0001^b^	< 0.0001^b^	0.0001^b^
56					
Control	86.3	86.0	67.7	33.7	24.3
Fluralaner	95.3	21.8	0	0	0
*P*-value		0.0276^b^	0.0006^b^	0.0082^b^	0.0165^b^
84					
Control	92.7	92.7	68.7	24.0	15.0
Fluralaner	94.8	78.0	1.5	1.0	1.0
*P*-value		0.2183	< 0.0001^b^	0.0019^b^	< 0.0001^b^

Student’s *t*-tests were performed to determine significant differences between groups. Regardless of treatment group, mosquito survival decreased upon infection with high numbers of microfilaria from infected dogs

^a^One non-treated dog stayed microfilaria-negative throughout, leading to improved mosquito survival; data from this dog was not included in the above calculations

^b^Indicates significant difference between control and fluralaner-treated mean live mosquito counts

**Table 3:** Percent survival (and 95% confidence intervals) of mosquitoes at 6 h, 24 h, 48 h, and 72 h after feeding on non-treated control (*n* = 3)^a^ or fluralaner-treated (*n* = 4) heartworm-infected, microfilaremic dogs

Days after treatment	6 h	24 h	48 h	72 h
2				
Control	99.0% (96.8–99.8)	66.2% (60.5–71.4)	14.3% (10.7–18.8)	9.1% (6.2–13.0)
Fluralaner	0% (0.0–1.1)	0% (0.0–1.1)	0% (0.0–1.1)	0% (0.0–1.1)
30				
Control	98.5% (96.1–99.6)	76.5% (71.1–81.1)	14.7% (11.0–19.4)	12.1% (8.7–16.6)
Fluralaner	3.8% (2.2–6.5)	0.6% (0.0–2.3)	0.3% (0.0–1.8)	0.3% (0.0–1.8)
56				
Control	99.6% (97.6–100.0)	78.4% (73.0–83.0)	39.0% (33.3–45.1)	28.2% (23.1–34.0)
Fluralaner	22.8% (18.9–27.3)	0% (0.0–1.2)	0% (0.0–1.2)	0% (0.0–1.2)
84				
Control	100% (98.4–100.0)	74.1% (68.6–78.9)	25.9% (21.1–31.4)	16.2% (12.3–21.0)
Fluralaner	82.3% (78.2–85.9)	1.6% (0.6–3.5)	1.1% (0.3–2.8)	1.1% (0.3–2.8)

^a^One non-treated dog stayed microfilaria-negative throughout, leading to improved mosquito survival; data from this dog were not included in the above calculations

**Table 4:** Live mosquitoes, mosquitoes with *Dirofilaria immitis* (*Di*) third-stage larvae, and mosquitoes with *D. immitis* PCR-positive counts 2 weeks after each mosquito challenge^a^

Challenge day	Number of live mosquitoes	Number with *Di* third-stage larvae	Number PCR-positive for *Di*
−7			
Control	19	3	13
Fluralaner	39	6	29
2			
Control	7	2	7
Fluralaner	0	NA	NA
30			
Control	8	3	5
Fluralaner	1	0	0
56			
Control	7	0	6
Fluralaner	0	NA	NA
84			
Control	12	1	12
Fluralaner	4	0	0

Day −7 was prior to treatment administration

*NA* not applicable

^a^One non-treated dog stayed microfilaria-negative throughout, leading to improved mosquito survival; data from this dog were not included in the above calculations

### Survival of mosquitoes fed on treated, microfilaremic dogs

For challenges following treatment, mean live counts of mosquitoes fed on fluralaner-treated dogs ranged from 0 to 78.0 6 h post-feeding with the highest survival count at the day-84 challenge. The remaining mosquito survival time points (24 h, 48 h, and 72 h post-feeding) had no more than 1.5 mosquitoes, on average, survive (Table 2). Two days after treatment, all (100%) mosquitoes fed on fluralaner-treated dogs were dead or severely moribund by 6 h post-feeding (Table 5) and therefore live counts and percent survival were zero for the remainder of the mosquito checks for this challenge (Tables 2 and 3). At 30 days post-treatment, 96.2% of mosquitoes fed on treated dogs were dead or severely moribund by 6 h after feeding, and > 99% were dead by 24 h (Table 5). At 56 days post-treatment, 77.2% of mosquitoes fed on treated dogs were dead or severely moribund by 6 h, and 100% were dead or severely moribund by 24 h. At 84 days post-treatment, 17.7% of mosquitoes fed on treated dogs were dead or severely moribund by 6 h, and > 98% were dead by 24 h. In summarizing the percent reduction (i.e., efficacy of treatment) of live mosquitoes fed on fluralaner-treated dogs, there was a 98.4–100% reduction by 24 h after feeding throughout the length of the study (Table 5). Two weeks after feeding on fluralaner-treated microfilaremic dogs, 0–4 mosquitoes survived, with the greatest number (four mosquitoes) surviving 2 weeks after the 84-day (12-week) post-treatment challenge (Table 4).

**Table 5:** Percent reduction in live mosquitoes at 6 h, 24 h, 48 h, and 72 h after feeding on fluralaner-treated (*n* = 4) heartworm-infected, microfilaremic dogs

Days after treatment	6 h (%)	24 h (%)	48 h (%)	72 h (%)
2	100	100	100	100
30	96.2	99.4	99.7	99.7
56	77.2	100	100	100
84	17.7	98.4	98.9	98.9

This was calculated by subtracting the percent survival from 100

When statistically comparing treated and control groups, mean live mosquito counts between the two groups were significantly different at every mosquito mortality check after each challenge except for the 6 h check at 84 days post-treatment (Table 2). Even though mosquito live counts gradually declined in each group, the differences between mean counts were significant in 15 of 16 comparisons (Table 2). Consistently significant differences were seen by the 24 h post-feeding check where 66.2–78.4% of mosquitoes fed on control dogs were alive in comparison to 0–1.6% of live mosquitoes after feeding on fluralaner-treated dogs (Table 3). Incidentally, and without quantification performed, videos were obtained 6 h after feeding to show immediately apparent survival differences between mosquitoes fed on control dogs and those fed on fluralaner-treated dogs (Additional file 1).

### Detection of *Dirofilaria immitis* in mosquitoes

Two weeks after feeding on microfilaremic dogs before treatment (day −7 challenge), assessment of surviving mosquitoes revealed that 15.5% (9/58; 95% CI 8.2–27.2) of the specimens had visible, motile third-stage larvae, and 72.4% (42/58; 95% CI 59.7–82.3) of the specimens were positive for *D. immitis* by PCR. Similarly, when assessing mosquitoes that survived 2 weeks after feeding on microfilaremic control dogs throughout the study, 17.7% (6/34; 95% CI 8.0–33.9) of the specimens had visible *D. immitis* third-stage larvae (Table 4), and 88.2% (30/34; 95% CI 72.8–95.9) were DNA-positive for *D. immitis* by PCR. When assessing the subset of mosquitoes which were divided by head and body, 18/23 (78.3%; 95% CI 57.7–90.8) were PCR-positive in at least one region, with 16.7% (3/18; 95% CI 5.0–40.1) of heads positive and 100% (18/18; 95% CI 79.3–100.0) of thorax/abdomen positive, suggesting that immature, arrested, or remnant larval stages were being detected. For three of the tested mosquitoes, both the head and thorax/abdomen had detectable *D. immitis* DNA. For mosquitoes which fed on fluralaner-treated dogs, only five survived 2 weeks after feeding throughout the study, and four of those five came from the last challenge (84 days post-treatment). However, none had visible third-stage larvae at dissection, and all were PCR-negative.

## Discussion

Isoxazoline efficacy against mosquitoes has been documented, and this approach has been proposed as a control strategy for mosquito-borne infections in the field [8, 11]. However, the present paper is one of a few published reports of in vivo mosquitocidal activity of an isoxazoline [29, 30]. A significant, pronounced reduction in mosquito survival was documented after feeding on fluralaner-treated dogs compared to mosquitoes fed on non-treated dogs (Table 2). This difference was significant at the earliest mosquito check time point—6 h after feeding—in all but the 84-day (12-week) post-treatment challenge. By 24 h after each feeding on fluralaner-treated dogs, 98% or greater of the mosquitoes were dead throughout the entire 84 days of the study. When compared to a similar study using afoxolaner, mosquitocidal efficacy 2 days after treatment was 98%, whereas fluralaner demonstrated 100% mosquito reduction in the current study at the same time point; additionally, nearly 1 month after treatment, the afoxolaner-treated dogs demonstrated efficacy at 75% but the present study resulted in 96% or greater mosquito reduction after feeding on fluralaner-treated dogs [29]. A separate study that utilized two different formulations of sarolaner for dogs demonstrated a wide range of efficacy against mosquitoes in comparison to the non-treated group across the study time frame [30]. For instance, at 24 h after feeding, mosquito efficacy ranged from −4.1% to 100%, but the lowest efficacy was noted 12 h after feeding and near the end of the study period (35 days post-treatment) [30]. These differences in onset and duration of efficacy between studies are likely due to the varying formulations across the isoxazoline drug class, and such differences should be considered when comparing and selecting a product for a particular target. Fluralaner is rapidly absorbed into the circulation to allow quick onset of action, and uniquely demonstrates extended efficacy due to its long half-life and high binding affinity to plasma proteins [6, 15].

Decreased survival was also seen in mosquitoes fed on non-treated dogs, likely due to very high microfilaremia present in infected dogs [31]. However, given the magnitude of the difference in mosquito survival between the fluralaner-treated and control dogs, a significant effect was still evident. Additionally, a video of mosquitoes immediately after feeding on fluralaner-treated dogs appears to document abnormal mosquito resting behavior (Additional File 2). Although not quantified, the tremors and fasciculations seen in mosquitoes fed on fluralaner-treated dogs suggest an immediate effect and warrant further investigation. Isoxazolines disrupt neurotransmission in invertebrates primarily by inhibiting gamma-aminobutyric acid (GABA)-gated channels, leading to hyperexcitation, paralysis, and insect death [32].

Of the mosquitoes that fed on microfilaremic fluralaner-treated dogs, very few survived 2 weeks after feeding in comparison to the non-treated group, and no *D. immitis* third-stage larvae were detected in mosquitoes fed on treated dogs. Even though fluralaner has a long elimination half-life and extended mean residence time, levels do wane over time [33]; this explains, in part, why most of the mosquitoes (4 out of 5) which survived 2 weeks after feeding on fluralaner-treated dogs were from the final mosquito feeding (day 84) when circulating fluralaner levels were likely at their lowest in the treatment group. Although classified as “fed” by visual inspection immediately after the challenge, these surviving mosquitoes may not have ingested a complete blood meal either coincidentally or due to behavior changes induced by early fluralaner exposure. Reduced ingestion of blood would be expected to limit the uptake of microfilaria and subsequent infection of mosquitoes as shown with topical insecticides [25]. Alternatively, the lack of *D. immitis* larvae or DNA in mosquitoes fed on fluralaner-treated dogs may be due to as-yet uncharacterized effects on the development of *D. immitis* in mosquitoes. However, there is currently no evidence to suggest isoxazolines exert a developmental effect, and further research is required to both confirm and more fully explain the apparent absence of *D. immitis* infection in mosquitoes fed on fluralaner-treated, microfilaremic dogs.

The experimental infections in the present study also provided an opportunity to monitor the development of *D. immitis* infection by different diagnostic tests. In the present study, dogs were first positive for *D. immitis* antigen with heat reversal 21 weeks (4.9 months) after inoculation, and without heat reversal 23–30 weeks (5.3–7 months) after inoculation, a similar time frame as previously reported [24, 34]. As expected from established infection models, circulating microfilaremia was confirmed in seven of eight dogs by 27 weeks (~ 6.3 months) after inoculation [21]. However, although evaluated for more than a year, circulating microfilaria never developed in one of the dogs. This dog apparently had an immune-mediated occult infection; plasma from the dog with occult infection caused in vitro clumping of microfilaria isolated from another dog (data not shown). Immune-mediated occult infections have long been recognized but are not considered common in experimentally infected dogs [35, 36]; explanations for occult infections include the development of antimicrofilarial antibodies and different cytokine expression profiles [37, 38]. Because assessment of microfilaria acquisition by mosquitoes fed on treated dogs was the primary objective of the current study, the amicrofilaremic dog was placed in the control group and not included in any comparisons or calculations as mosquitoes fed on this dog showed, at times, significantly higher post-feeding survival in comparison to those fed on microfilaremic dogs (data not shown).

## Conclusion

Fluralaner treatment of heartworm-positive dogs at the label-approved dose results in potent mosquitocidal effects for as long as 12 weeks. Since mosquitoes that feed on heartworm-infected dogs that receive fluralaner every 12 weeks would die before the development or transmission of infective *D. immitis* larvae could occur, this regular treatment can potentially limit transmission of heartworm between dogs in a community. This strategy may be particularly important to consider when managing a dog infected with a known macrocyclic lactone-resistant isolate of *D. immitis*. Indeed, the integration of fluralaner into the treatment plan could be a key factor in preventing the spread of canine heartworm in communities.

## Supplementary Information

**Additional file 1: **Video of mosquitoes 6 h after feeding on control dogs (first cup) and mosquitoes fed on fluralaner-treated dogs (second cup).

**Additional file 2: **Videos of female *Aedes aegypti* (Liverpool) immediately after feeding on heartworm-positive dogs **A** given fluralaner or **B** left untreated, 2 days earlier.

## Data Availability

Data supporting the conclusions of this article are included within the article. The summary datasets used and analyzed are available from the corresponding author upon reasonable request.
